# Biochemical Characteristics of Urine Metabolomics in Female Giant Pandas at Different Estrous Stages

**DOI:** 10.3390/ani14233486

**Published:** 2024-12-03

**Authors:** Donghui Wang, Jiasong Chen, Shili Wu, Kailai Cai, Junhui An, Mingyue Zhang, Xiangwei Kong, Zhigang Cai, Yuan Li, Hongyan Li, Cuiyu Long, Yijiao Chen, Rong Hou, Yuliang Liu, Jingchao Lan

**Affiliations:** 1 Chengdu Research Base of Giant Panda Breeding, Chengdu 610081, China; wangdh889@163.com (D.W.);; 2 Sichuan Key Laboratory of Conservation Biology for Endangered Wildlife, Chengdu 610081, China; 3 Sichuan Academy of Giant Panda, Chengdu 610081, China

**Keywords:** giant panda, urine, metabolites analysis, estrus monitoring

## Abstract

The composition of urinary metabolites can reflect the physiological state of animals. Investigating the alterations in urine metabolomics during the estrus stage can provide valuable insights for enhancing the efficacy of estrus monitoring. This study aimed to perform an analysis of urinary metabolomics in female giant pandas, specifically examining the variations in specific metabolites across different estrous stages, namely, diestrus, proestrus, estrus, and metestrus. A total of 1234 metabolites were identified in positive ion mode from 76 samples of 19 individuals, with 643 metabolites identified in negative ion mode. The content of urine metabolites exhibited significant variation throughout different stages of estrus. During the peak of estrus, the metabolic pathways primarily enriched by significantly differential metabolites were the AMPK signaling pathway, vitamin digestion and absorption, galactose metabolism, and cysteine and methionine metabolism, as well as taurine and hypotaurine metabolism. By comparing the content of specific metabolites in distinct pathways across the four distinct estrous stages, higher levels of acetylcholine, D-fructose1,6-bisphosphate, L-homocystine, dulcitol, inositol, and S-sulfo-L-cysteine and lower levels of phosphoethanolamine, vitamin A, vitamin B12, and maleic acid were detected at estrus. This study offers a novel comparative analysis of urine metabolomics across different estrus stages in female giant pandas, identifying several potential perspectives for estrus monitoring and contributing to the breeding management of captive giant panda populations.

## Simple Summary

Urine metabolomics analysis is a valuable tool for assessing the organism’s metabolic status, and it plays a crucial role in elucidating health conditions, physiological changes, disease progression, and environmental adaptation. Presently, this technology is widely employed in human clinical disease diagnosis, as well as livestock breeding and selection. This study aimed to perform an analysis of urinary metabolomics in female giant pandas at different estrous stages. The results showed that the content of urine metabolites exhibited significant variation throughout different stages of estrus. During estrus, there were higher levels of acetylcholine, D-dructose1,6-bisphosphate, L-homocystine, dulcitol, inositol, and S-sulfo-L-cysteine, which were detected in a manner consistent with fluctuations in estrogen levels. Conversely, there were lower levels of phosphoethanolamine, vitamin A, vitamin B12, and maleic acid in a manner consistent with changes in progesterone levels. This study found several potential perspectives for estrus monitoring and contributing to the breeding management of captive giant panda populations.

## 1. Introduction

The giant panda (*Ailuropoda melanoleuca*) is a vulnerable species that is native to China. During the 18th and 19th centuries, giant pandas were extensively distributed in many provinces of China including Sichuan, Shaanxi, Hunan, Hubei, Guizhou, and Yunnan provinces [1,2]. Due to different degrees of habitat fragmentation and degradation, some local wild giant pandas populations still face survival risks. Currently, the wild population is constrained to an estimated 1864 individuals residing within fragmented bamboo forests spanning 25,800 km^2^ across six mountain ranges in southwestern China (https://www.forestry.gov.cn/c/www/lczy/38173.jhtml (accessed on 13 August 2023)). In order to preserve rare species of mammals, constant monitoring is carried out in the world’s mammal fauna, and various strategies are implemented to preserve their populations [3,4]. Specific methods for effective study and conservation are known for different mammal species. The ex situ conservation efforts for giant pandas have led to a notable increase in their population, thereby contributing significantly to the biodiversity conservation of this species. The acquisition of these achievements is inseparable from the ongoing advancements in artificial breeding technology. Historically, the reproduction of giant pandas has been difficult and continues to be challenging [5]. One of the most important reasons is that female giant pandas experience only one estrus period per year, which lasts for a very brief duration of usually 2–3 days. Therefore, precise monitoring of the estrus status to pinpoint the timing of ovulation is crucial for the success of both timed mating and artificial insemination.

Currently, the main methods for estrus monitoring in female giant pandas include behavioral observation, such as appetite changes, mental state, and changes in the odor of urine [6]; vaginal cytology and vulvar swelling [7]; and monitoring of endocrine levels [8]. Monitoring the fluctuations in urinary estrogen and progesterone levels is a widely employed method for assessing estrus in giant pandas and forecasting optimal mating times. Typically, during the estrus peak, estrogen levels reach the peak and then decline, whereas progesterone levels begin to ascend from baseline values [9,10]. The ideal time for mating is determined through a comprehensive analysis of the trajectories of these hormonal changes. Nonetheless, there are instances where certain individuals exhibit atypical patterns in estrogen or progesterone fluctuations, complicating the accurate determination of the estrus and mating time.

In recent years, urinary metabolomics analysis has become a method of understanding the physiological status of an organism by analyzing the combination of metabolites in urine [11], leading to insights into disease progression, environmental adaptation, and physiological changes [12,13,14]. Metabolomics analysis of urine offers numerous advantages and potential applications in animal research. The simplicity and non-invasiveness of urine collection minimizes impacts on humans or animals and facilitates large-scale sample collection [15,16]. A study conducted on mice demonstrated that proteins and certain fatty acids increase during the estrus phase and might serve as urinary biomarkers for the detection of estrus [17]. The untargeted metabolomics analysis of buffalo urine identified a significant upregulation of hydracrylic acid, 3-bromo-1-propanol, and benzyl serine during the estrus phase, offering valuable biomarkers for determining estrus in buffalo [18]. Variations in the concentrations of specific biochemical constituents in urine across different estrous cycles are dependent on the stage of the animal and its hormonal status [17]. Therefore, it can be hypothesized that the urinary metabolites at various estrus stages include those directly associated with estrogen or progesterone, as well as metabolites that are not regulated by these hormones. Identifying these metabolites could potentially address the limitations of assessing the estrus cycle in giant pandas solely through estrogen and progesterone levels.

Urine metabolomics research has great application prospects and value in the field of endangered wildlife conservation. Recent research has identified differential metabolites between non-pregnant and pregnant states in Yangtze finless porpoises, with a focus on amino acid and carbohydrate metabolism. These findings suggest that metabolic activity varies throughout different stages of pregnancy and that levels of urine metabolites may serve as indicators for pregnancy in this species [19]. The urinary metabolomics of giant pandas at different stages of pregnancy exhibited significant variations, and changes in specific urinary metabolites may provide valuable support for determining pregnancy in giant pandas [20].

Accordingly, we proposed a hypothesis that there might be metabolites present in the urine of female giant pandas that could potentially function as metabolic biomarkers for the estrus onset. Therefore, in this study, we aimed to examine the dynamic changes in urinary metabolites among female giant pandas during different phases of the estrous cycle, which might provide a potential method for determining the estrus status of female giant pandas. These valuable data will enhance our understanding of the underlying mechanisms governing reproduction in giant pandas and may have implications for conservation efforts and captive breeding programs.

## 2. Materials and Methods

### 2.1. Animals and Urine Samples

**Figure 1:**
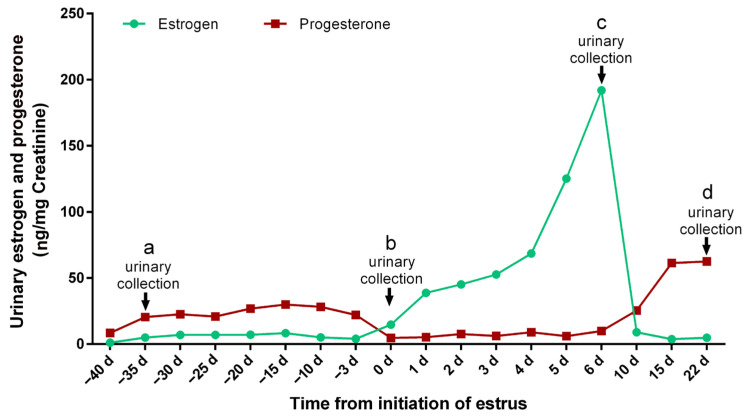
Typical urinary estrogen and progesterone profiles during estrous periods. The black arrows indicate the time to collect the corresponding urine sample. The letters a, b, c, and d represent the diestrus, proestrus, estrus, and metestrus groups, respectively.

In this study, 19 sexually mature female giant pandas with a mean body weight of 106.4 ± 10.7 kg, feeding in the Chengdu Research Base of Giant Panda Breeding, Chengdu, Sichuan province, China, were investigated (Table S1). All the giant pandas participated in the breeding program in 2023. During the breeding season, the estrogen and progesterone levels in urine samples of female giant pandas were monitored. The whole estrous cycle was divided into four phases (diestrus, proestrus, estrus, and metestrus) according to the hormones level [21] (Figure 1). The urine sample collection was performed as previously reported [20]. Briefly, urine samples (about 2–3 mL each) were collected from giant pandas during the four phases of the estrous cycle. The urine samples were collected from a clean cement floor with 10 mL sterile syringes, then transferred to 15 mL centrifuge tubes (Corning Incorporated, Corning, NY, USA) and labeled with the animal’s name and the date of collection. All the samples were stored at −80 °C until analysis. Urine samples (collected between 6 AM and 8 AM) were selected for UHPLC-MS/MS analyses.

### 2.2. Samples Processing

The urine samples (100 μL) were resuspended with prechilled 80% methanol by a well vortex. Then the samples were incubated on ice for 5 min and centrifuged at 15,000× *g*, 4 °C, for 20 min. The supernatant was diluted to the final concentration containing 53% methanol by LC-MS grade water. The samples were subsequently transferred to a clean tube and then were centrifuged at 15,000× *g*, 4 °C, for 20 min. Finally, the supernatant was injected into the LC-MS/MS system analysis [1,2]. Pooled quality control (QC) samples were prepared by mixing 10 µL from each sample supernatant [22].

### 2.3. UHPLC-MS/MS Analysis

UHPLC-MS/MS analyses were performed using a Vanquish UHPLC system (Thermo Fisher Scientific, Bremen, Germany) coupled with an Orbitrap Q ExactiveTM HF-X mass spectrometer (Thermo Fisher Scientific, Bremen, Germany) in Novogene Co., Ltd. (Beijing, China). Samples were injected onto a Hypersil Gold C18 column (100 × 2.1 mm, 1.9 μm, Thermo Fisher Scientific, Bremen, Germany) using a 17 min linear gradient at a flow rate of 0.2 mL/min. The column temperature was maintained at 35 °C. The eluents for the positive polarity mode were eluent A (0.1% formic acid in water) (Sigma, St. Louis, MO, USA) and eluent B (methanol) (Sigma). The eluents for the negative polarity mode were eluent A (5 mM ammonium acetate, pH 9.0) (Sigma) and eluent B (methanol) [23]. The solvent gradient was set as follows: 2% B, 1.5 min; 2–85% B, 3 min; 85–100% B, 10 min; 0–2% B, 10.1 min; and 2% B, 12 min. The Q ExactiveTM HF mass spectrometer was operated in positive/negative polarity mode with a spray voltage of 3.5 kV, a capillary temperature of 320 °C, a sheath gas flow rate of 35 psi, an aux gas flow rate of 10 L/min, an S-lens RF level of 60, and an aux gas heater temperature of 350 °C.

### 2.4. Data Processing and Metabolite Identification

The raw data files generated by UHPLC-MS/MS were processed using the Compound Discoverer 3.3 (CD3.3) (Thermo Fisher Scientific, Waltham, MA, USA) to perform peak alignment, peak picking, and quantitation for each metabolite. The main parameters were set as follows: the peak area was corrected with the first QC samples, actual mass tolerance, 5 ppm; signal intensity tolerance, 30%; and minimum intensity. Then, peak intensities were normalized to the total spectral intensity. The normalized data were used to predict the molecular formula based on additive ions, molecular ion peaks, and fragment ions. Then peaks were matched with the mzCloud (https://www.mzcloud.org/ (accessed on 10 July 2023)), mzVault, and MassList databases to obtain the accurate qualitative and relative quantitative results. Statistical analyses were performed using the statistical software R (R version R-3.4.3), Python (Python 2.7.6 version), and CentOS (CentOS release 6.6). When data were not normally distributed, the area normalization method was employed for standardizing the transformation.

### 2.5. Data Analysis

Metabolites were annotated using the Kyoto Encyclopedia of Genes and Genomes (KEGG) database (https://www.genome.jp/kegg/pathway.html (accessed on 10 July 2023), the Human Metabolome Database (http://www.hmdb.ca (accessed on 10 July 2023)), and the LIPID MAPS database (http://www.lipidmaps.org/ (accessed on 10 July 2023)). The principal component analysis (PCA) and partial least squares discriminant analysis (PLS-DA) were performed with metaX [24]. The statistical significance (*p*-value) was calculated by univariate analysis (*t*-test). The metabolites were considered to have significant differences with the variable importance in projection (VIP) > 1 and *p*-value < 0.05 and fold change (FC) ≥ 2 or FC ≤ 0.5. For clustering heat maps, the data of the intensity areas of differential metabolites were normalized using the z-score formula, Value = [(Value) − Mean(Row)]/[Standard deviation(Row)], and plotted by the Pheatmap package in R. The correlations between differential metabolites were analyzed by cor () in R (method = Pearson). The statistical significances of correlations between differential metabolites were calculated by cor.mtest () in R. A *p*-value < 0.05 was considered as statistically significant, and correlation plots were plotted by the corrplot package in R. The functions of these metabolites and metabolic pathways were studied using the KEGG database. The metabolic pathways of differential metabolites were considered as enrichment when the ratio was satisfied by x/n > y/N, and metabolic pathways were considered as statistically significant enrichment when the *p*-value < 0.05.

## 3. Results

### 3.1. Untargeted Metabolic Profiling of Urine at Different Estrous Stages

**Figure 2:**
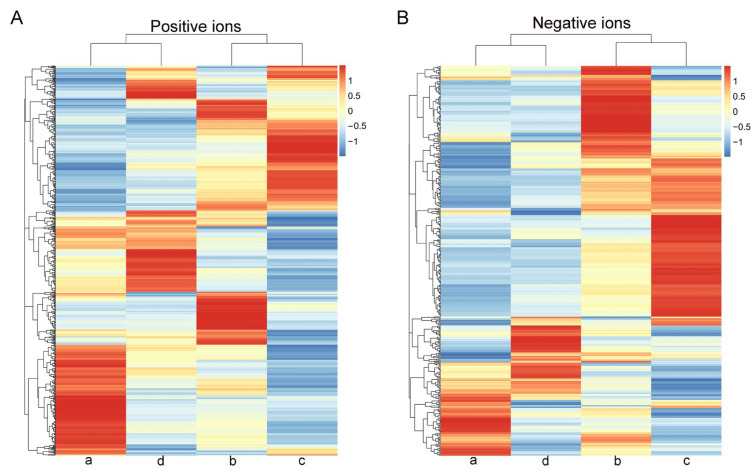
A heat map of the metabolites identified in the urine of different estrous stages of 19 female giant pandas. (**A**) Positive ions. (**B**) Negative ions. The letters a, b, c, and d represent the diestrus, proestrus, estrus, and metestrus groups, respectively.

To investigate the metabolic changes in urine at different estrous stages in giant pandas, non-targeted metabolomics analysis was performed. In the positive and negative ion modes, the correlations of QC samples and R2 values were close to 1 (Figure S1). In this study, a total of 1234 metabolites were identified in positive ion mode from 76 samples, and 643 metabolites were identified in negative ion mode. The cluster analysis diagram both in positive and negative ions mode showed that the differential metabolites in the “a” and “d” groups were clustered, and “b” and “c” groups were clustered (Figure 2).

### 3.2. Multivariate Statistical Analysis

**Figure 3:**
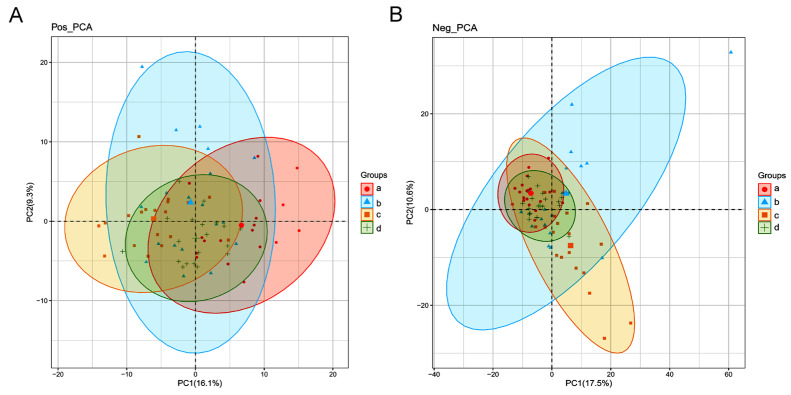
The PCA score plots of metabolites identified in the urine of different estrous stages of female giant pandas. (**A**) Positive ions. (**B**) Negative ions. Each point on the PCA score chart represents a sample. The letters a, b, c, and d represent the diestrus, proestrus, estrus, and metestrus groups, respectively.

The PCA was performed to assess the sample separation and aggregation among the four different estrous stages of female giant pandas. Aggregation of points indicated that the observed variables were highly similar, and discrete points represented significant differences in the observed variables (VIP ≥ 1; ratio ≥ 2 or ratio ≤ 1/2; q ≤ 0.05). In the positive ion mode, the PCA score showed 16.1% variation at PC1 and 9.3% variation at PC2 (Figure 3A). In the negative ion mode, the PCA score showed 17.5% variation at PC1 and 10.6% variation at PC2 (Figure 3B).

**Figure 4:**
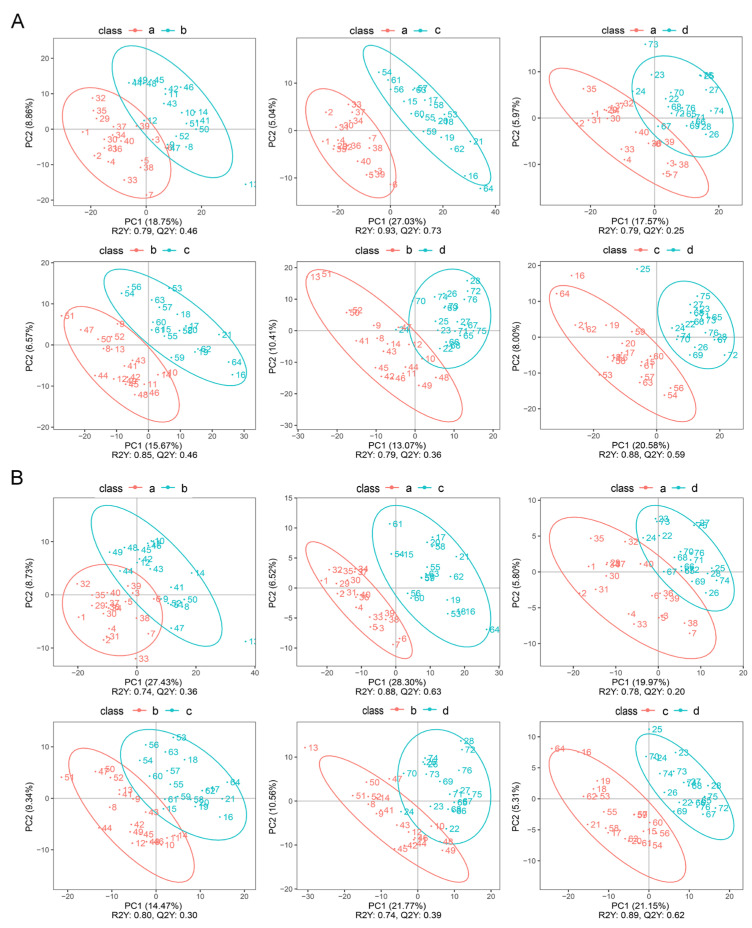
The PLS-DA chart. (**A**) Positive ions. (**B**) Negative ions. The letters a, b, c, and d represent the diestrus, proestrus, estrus, and metestrus groups, respectively.

The results of PLS-DA showed that in positive ion mode, for the comparative analysis of the diestrus and proestrus, R2 = 0.79 and Q2 = 0.46; for the comparative analysis of the diestrus and estrus, R2 = 0.93 and Q2 = 0.73; for the comparative analysis of the diestrus and metestrus, R2 = 0.79 and Q2 = 0.25; for the comparative analysis of the proestrus and estrus, R2 = 0.85 and Q2 = 0.46; for the comparative analysis of the proestrus and metestrus, R2 = 0.79 and Q2 = 0.36; and for the comparative analysis of the estrus and metestrus, R2 = 0.88 and Q2 = 0.59 (Figure 4A).

In negative ion mode, for the comparative analysis of the diestrus and proestrus, R2 = 0.74 and Q2 = 0.36; for the comparative analysis of the diestrus and estrus, R2 = 0.88 and Q2 = 0.63; for the comparative analysis of the diestrus and metestrus, R2 = 0.78 and Q2 = 0.20; for the comparative analysis of the proestrus and estrus, R2 = 0.80 and Q2 = 0.30; for the comparative analysis of the proestrus and metestrus, R2 = 0.74 and Q2 = 0.39; and for the comparative analysis of the estrus and metestrus, R2 = 0.89 and Q2 = 0.62 (Figure 4B).

### 3.3. Metabolic Pathways for Metabolite Analysis

**Figure 5:**
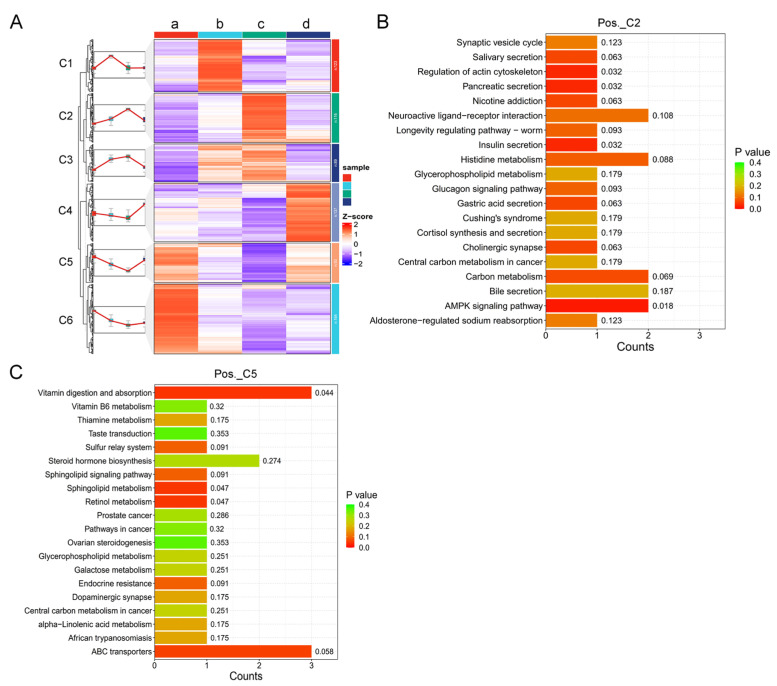
Analysis of the differential metabolites of positive ions in the urine of different estrous stages of female giant pandas. (**A**) Heat map of the differential metabolites in the four estrous stages. The letters a, b, c, and d represent the diestrus, proestrus, estrus, and metestrus groups, respectively. (**B**) The KEGG enrichment map of cluster 2 (Pos._C2). (**C**) The KEGG enrichment map of cluster 5 (Pos._C5).

In order to search for potential metabolite markers in the urine of female giant pandas during estrus, we performed clustering analysis on differential metabolites at four different estrous stages. The differential metabolites were clustered into six clusters in positive ion mode (Figure 5A). Then, we conducted a KEGG analysis on the two clusters in positive ion mode, which showed the most significant changes in metabolites during estrus. In cluster 2 (Pos._C2), the significant differences were in the AMPK signaling pathway (*p* < 0.05), regulation of actin cytoskeleton (*p* < 0.05), insulin secretion (*p* < 0.05), and pancreatic secretion (*p* < 0.05) (Figure 5B). In cluster 5 (Pos._C5), the significant differences were in the vitamin digestion and absorption (*p* < 0.05), sphingolipid metabolism (*p* < 0.05), and retinol metabolism (*p* < 0.05) (Figure 5C).

**Figure 6:**
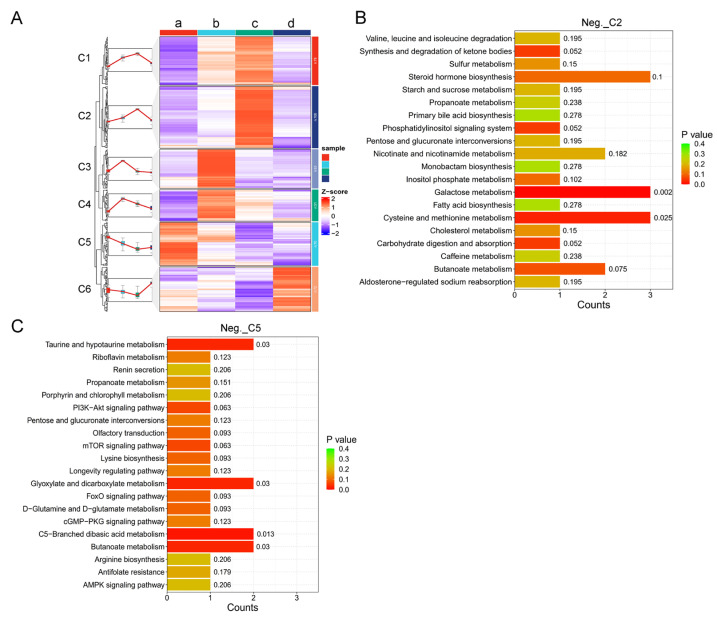
Analysis of the differential metabolites of negative ions in the urine of different estrous stages of female giant pandas. (**A**) Heat map of the differential metabolites in the four estrous stages. The letters a, b, c, and d represent the diestrus, proestrus, estrus, and metestrus groups, respectively. (**B**) The KEGG enrichment map of cluster 2 (Neg._C2). (**C**) The KEGG enrichment map of cluster 5 (Neg._C5).

In negative ion mode, differential metabolites at four different estrous stages were also clustered into six clusters (Figure 6A). Similarly, we conducted the KEGG analysis on the two clusters in negative ion mode that showed the most significant changes in metabolites during estrus. In cluster 2 (Neg._C2), the significant differences were in the galactose metabolism (*p* < 0.05), cysteine, and methionine metabolism (*p* < 0.05) (Figure 6B). In cluster 5 (Neg._C5), the significant differences were in the C5-Branched dibasic acid metabolism (*p* < 0.05), taurine and hypotaurine metabolism (*p* < 0.05), glyoxylate and dicarboxylate metabolism (*p* < 0.05), and butanoate metabolism (*p* < 0.05) (Figure 6C).

### 3.4. Content Characteristics of the Potential Biomarkers Metabolites

**Figure 7:**
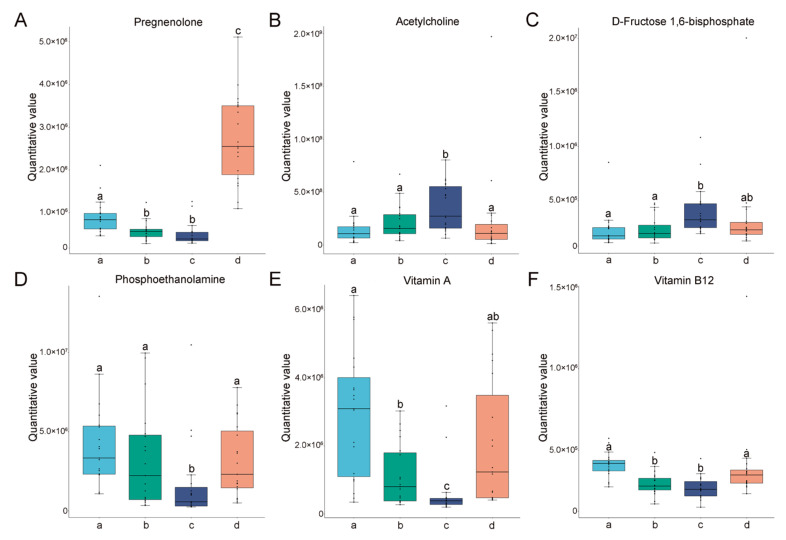
The changes and differences in differential metabolites in positive ions among different groups. The y-axis represents the quantitative values of metabolites, and the x-axis represents different groups. (**A**) Pregnenolone. (**B**) Acetylcholine. (**C**) D-fructose 1,6-bisphosphate. (**D**) Phosphoethanolamine. (**E**) Vitamin A. (**F**) Vitamin B12. The letters a, b, c, and d in the X-axis represent the diestrus, proestrus, estrus, and metestrus groups, respectively. Columns without common letters indicate statistical difference (*p* < 0.05 for (**B**,**D**,**F**), *p* < 0.01 for (**A**,**C**,**E**)).

**Figure 8:**
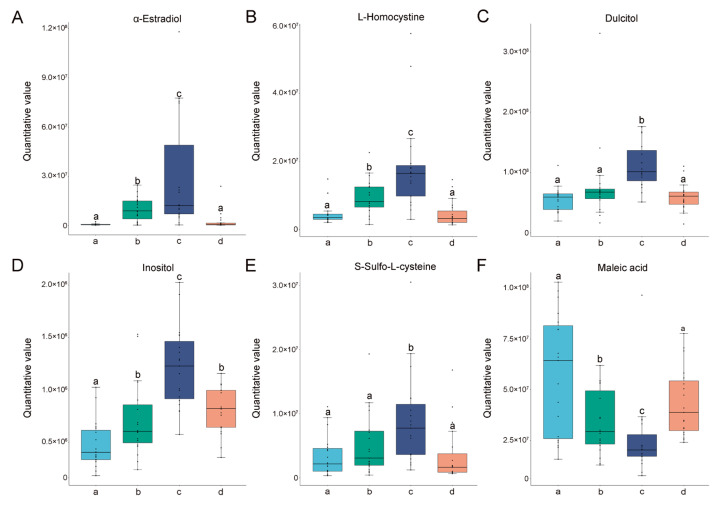
The changes and differences in differential metabolites in negative ions among different groups. The y-axis represents the quantitative values of metabolites, and the x-axis represents different groups. (**A**) α-estradiol. (**B**) L-homocystine. (**C**) Dulcitol. (**D**) Inositol. (**E**) S-sulfo-L-cysteine. (**F**) Maleic acid. The letters a, b, c, and d on the X-axis represent the diestrus, proestrus, estrus, and metestrus groups, respectively. Columns without common letters indicate statistical difference (*p* < 0.05 for (**A**,**B**,**D**–**F**), *p* < 0.01 for (**C**)).

Furthermore, we compared the content of specific metabolites in the significantly different pathways in Pos._C2 and Pos._C5 among the four different estrous stages. The contents of acetylcholine (Figure 7B) and D-fructose 1,6-bisphosphate (Figure 7C) were gradually increased from the proestrus then reached a peak during estrus, followed by a decrease during metestrus. On the contrary, phosphoethanolamine (Figure 7D), vitamin A (Figure 7E), and vitamin B12 (Figure 7F) were at the lowest levels at estrus. Moreover, the contents of specific metabolites in the significantly different pathways in Neg._C2 and Neg._C5 among the four different different estrous stages were also compared. The contents of L-homocystine (Figure 8B), dulcitol (Figure 8C), inositol (Figure 8D), and S-sulfo-L-cysteine (Figure 8E) were researched the highest point at estrus. Oppositely, the maleic acid was at the lowest level at estrus (Figure 8F). At the same time, two widely recognized hormonal markers of estrus, pregnenolone (Figure 7A) and α-estradiol (Figure 8A), were also detected, and their changing trends were in accordance with normal patterns, reflecting the reliability of our detection.

## 4. Discussion

In this study, we conducted a characterization of the urinary metabolome from 19 female giant pandas. The estrus expression of all individuals fell within normal parameters, with consistent changes observed in urinary hormone levels. The sample size collected met the necessary criteria for statistical analysis. Longitudinal metabolome variation associated with different stages of estrus was investigated. And by comparing the content of specific metabolites in distinct pathways across the four distinct estrous stages, acetylcholine, D-fructose 1,6-bisphosphate, L-homocystine, dulcitol, inositol, S-sulfo-L-cysteine, phosphoethanolamine, vitamin A, vitamin B12, and maleic acid may be candidate biomarkers for estrus monitoring. This study provides the basic urine metabolome characteristics of different stages of estrus for further estrus prediction research in giant pandas.

In the present study, through cluster analysis, both positive ion and negative ion modes showed clustering of dioestrus and metestrus and clustering of proestrus and estrus (Figure 2). These results demonstrated that the urine metabolites of giant pandas were highly sensitive to estrus changes and changed greatly after estrus initiation, which might be a potential reference to indicate the stage of estrus. In addition, the results of PCA analysis also revealed that there were more pronounced changes in metabolites in both the proestrus phase and the estrus phase in the negative ion mode (Figure 3). These results suggested that certain metabolic pathways or compounds related to these phases were more prominently represented in the negative ion mode. Furthermore, the results of PLS-DA also indicated that metabolite changes in urine were significant during different stages of estrus (Figure 4). Similarly, the previous study showed that urine biochemical constituent concentrations vary depending on the reproductive cycle phase and hormonal status of the animals and may contribute to variation across reproductive cycles [17].

The estrous cycle is a continuous physiological process, and there is a close relationship between different stages of estrus. From the perspective of urine metabolomics, it is imperative to analyze the dynamic trends of specific metabolites at different stages in order to ascertain the estrus phase in animals. Only through examination of the content changing patterns of a particular metabolite throughout the entire estrus phase can we assess its potential as a viable biomarker for estrus. Consequently, our investigation on alterations in urine metabolites during the complete estrous cycle of giant pandas specifically focused on metabolites exhibiting higher and lower levels during the peak estrus period, followed by regulatory signal pathway analysis. The results revealed that metabolites with higher levels during this critical stage were predominantly enriched in AMPK signaling pathway, regulation of actin cytoskeleton, insulin secretion, pancreatic secretion, galactose metabolism, and cysteine and methionine metabolism pathways; those with lower levels were primarily associated with vitamin digestion and absorption sphingolipid metabolism, retinol metabolism, C5-branched dibasic acid metabolism, taurine and hypotaurine metabolism, glyoxylate and dicarboxylate metabolism, and butanoate metabolism pathways (Figure 5 and Figure 6).

Furthermore, we focused on key metabolites that underwent significant changes in metabolic pathways and conducted a quantitative analysis of these metabolites at different stages of estrus. In positive ion mode, we first analyzed the trend of pregnenolone. It is widely acknowledged that the pregnenolone increases significantly during the metestrus [8], which is consistent with our results and supports the accuracy of the measurements. Acetylcholine and D-fructose 1,6-bisphosphate showed the highest levels during the peak estrus phase and may serve as potential biomarkers in the positive mode of metabolite analysis. Acetylcholine has been shown in mouse models to regulate steroid synthesis and apoptosis in the ovaries, as well as to regulate reproductive endocrine functions [25]. Research conducted on rats has demonstrated that elevated estrogen levels stimulate the secretion of acetylcholine in the brain [26]. Consequently, the increasement of acetylcholine during the peak estrus stage can be explained by the heightened estrogen levels at this time, thereby underscoring the reliability of this phenomenon. A high-energy glycolytic intermediate, fructose 1,6-bisphosphate, has been shown to protect a variety of cell types and tissues from harmful substances [27]. Interestingly, recent studies have demonstrated that comparative proteomic analysis of water buffalo saliva reveals a significant enrichment of the glycolysis signaling pathway during estrus, indicating a crucial regulatory role of this metabolic pathway in governing the process of the estrous cycle [28]. Therefore, D-fructose 1,6-bisphosphate might be a new potential indicator of estrus in female giant pandas. In addition, we also found that phosphoethanolamine, vitamin A, and vitamin B12 were the lowest at estrus. There are relatively few studies in this area, and there are only reports that excess vitamin A inhibits estrus [29], which may explain why vitamin A levels were minimized during peak estrus in giant pandas. In addition, studies in humans have reported that oral estrogen significantly reduces the level of vitamin B12 in plasma [30], and this might explain why the content of vitamin B12 reached the lowest level during the peak estrus phase. It is also worth noting that dietary intake is also one of the most important factors influencing changes in urinary metabolites in giant pandas, and there are significant changes in feeding behavior during estrus. Thus, in the present study, we only focused on the metabolite trends to guide the identification of the estrous phase, and the deeper mechanisms need further study.

In negative ion mode, the expression trend of α-estradiol was first analyzed, which confirmed the reliability of the test results. Then, in our results, the relative levels of inositol, S-sulfo-L-cysteine, L-homocystine, and dulcitol reached the highest point at estrus, suggesting that these metabolites may be involved in the regulation process of giant panda estrus. First, inositol is an essential carbocyclic sugar polyalcohol for signal transduction, metabolic flux, insulin signaling, ion-channel permeability regulation, and embryonic development [31]. In addition, it was found that increased serum inositol levels were positively correlated with estrus rate in yaks [32]. The detection outcomes of metabolites in the oviductal fluid of Holstein heifers during the day 3 post-estrus phase indicated that the contents of lactic acid, glycine, and inositol were the highest [33]. These published studies could support our results. Second, S-Sulfo-L-cysteine is derived from cysteine and methionine metabolism. An increase in the cysteine content of a tissue may indicate an increase in the metabolic activity of that tissue [34]. Furthermore, it has been shown that the presence of glutathione in the ovary is an important defense mechanism against oxidative damage, while glutathione in oocytes plays a crucial role in early embryonic development [35,36]. The biosynthesis of glutathione mainly depends on cysteine and methionine metabolism [37,38]. At the peak of estrus in female giant pandas, ovarian follicular metabolic activity is intense and generates large ROS accumulation, thus requiring cysteine metabolism regulation. In addition, other findings were observed in spermatozoa: a significant increase in S-sulfo-L-cysteine content in activated spermatozoa may be a result of sperm self-protection mechanisms to minimize sperm damage caused by large amounts of ROS during activation. Thus, S-sulfo-L-cysteine may be expected to be a potential estrus recognition marker for giant pandas. Similarly, dulcitol was also able to increase glutathione levels in oocytes and positively affect oocyte maturation [39]. In vitro mass spectrometry studies showed that estrogen could form conjugates with L-homocysteine and modulate the level of L-homocystine [40]. Elevated levels of estrogen suggest a potential for increased interaction with L-homocysteine, thereby indicating that L-homocysteine might serve as a urine metabolic marker for the estrus stage, analogous to estrogen.

Previous studies have shown that the addition of maleic acid to human and golden gopher spermatozoa reduces their viability and penetration capacity and even rapidly leads to spermicidal effects [41,42]. In addition, synthetic polymers based on maleic acid have also shown potent effects in inhibiting sperm motility [43]. In our results, the content of maleic acid metabolites was significantly reduced during peak estrus. This phenomenon may be explained by the fact that maleic acid is strongly spermicidal. When female giant pandas ovulate and are ready to conceive at the peak of estrus, a significant reduction in maleic acid content could help to improve sperm survival after mating and increase conception success.

## 5. Conclusions

Urine metabolomics analysis exhibits high regularity and reproducibility, holding great potential for long-term monitoring and evaluation of biological changes. This non-invasive method not only provides accurate estrus monitoring but also strengthens the understanding of physiological changes and reproductive biology in animals. In this study, we examined and analyzed the changes of urine metabolomics in the complete reproductive cycle of female giant pandas for the first time. Through the changes of metabolites in different estrus stages, we originally explored several potential markers, such as acetylcholine, D-fructose 1,6-bisphosphate, L-homocystine, dulcitol, inositol, S-sulfo-L-cysteine, phosphoethanolamine, vitamin A, vitamin B12, and maleic acid, which might indicate the estrus onset. This study laid an important foundation for estrus monitoring female giant pandas and potentially aiding in the breeding management of captive giant panda populations.

## Data Availability

Data are contained within the article and Supplementary Materials.

## Supplementary Materials

The following supporting information can be downloaded at https://www.mdpi.com/article/10.3390/ani14233486/s1, Figure S1. The quality control. (A) Chart positive ions. (B) Chart negative ions.; Table S1. Urine samples collection from female giant pandas; Table S2. The specific metabolites in Pos_cluster 2; Table S3. The specific metabolites in Pos_cluster 5; Table S4. The specific metabolites in Neg_cluster 2; Table S5. The specific metabolites in Neg_cluster 5.
