# Supplementary material for: Biochemical Characteristics of Urine Metabolomics in Female Giant Pandas at Different Estrous Stages
Source: Animals (Basel). 2024 Dec 3;14(23):3486. doi: 10.3390/ani14233486 (PMC11640436; doi:10.3390/ani14233486)
Supplement: Supplementary file 1 [file animals-14-03486-s001.zip › Table S1. Urine samples collection from female giant pandas.pdf]

Table S1. Urine samples collection from female giant pandas.

Studbook number	Age	Estrous behavior	Stress resistance	Date of sample collection	Estrogen ng/mg Creatinine	Progesterone ng/mg Creatinine
762	14	Normal estrus behavior	No obvious stress pressure	2022/12/7	1.58	11.02
				2022/12/31	10.86	6.19
				2023/1/7	98.35	12.74
				2023/1/24	2.95	56.49
966	8	Normal estrus behavior	No obvious stress pressure	2023/1/14	3.65	18.63
				2023/1/23	14.79	9.44
				2023/2/1	101.36	7.72
				2023/3/2	3.29	47.49
665	16	Normal estrus behavior	No obvious stress pressure	2022/12/20	1.80	21.13
				2023/2/1	21.16	5.20
				2023/2/7	133.38	14.18
				2023/2/23	3.75	64.63
635	17	Normal estrus behavior	No obvious stress pressure	2023/1/1	5.01	20.55
				2023/2/6	14.72	4.68
				2023/2/12	191.96	9.90
				2023/2/28	4.94	41.51
824	12	Normal estrus behavior	No obvious stress pressure	2023/1/6	7.28	18.29
				2023/2/10	14.31	9.66
				2023/2/19	185.81	11.03
				2023/2/26	4.11	58.17
980	8	Normal estrus behavior	No obvious stress pressure	2023/1/14	4.54	16.59
				2023/2/13	22.68	1.61
				2023/2/19	159.54	14.78
				2023/3/23	1.88	48.01

765	17	Normal estrus behavior	No obvious stress pressure	2023/2/9	3.26	27.82
				2023/3/5	11.96	40.93
				2023/3/19	158.44	9.71
				2023/3/29	3.18	41.84
990	13	Normal estrus behavior	No obvious stress pressure	2023/3/2	3.88	17.63
				2023/3/15	19.02	20.36
				2023/3/27	169.73	10.83
				2023/5/9	8.37	125.85
965	8	Normal estrus behavior	No obvious stress pressure	2022/12/12	1.26	9.66
				2022/12/31	10.92	4.22
				2023/1/9	155.20	9.80
				2023/2/17	5.53	82.94
1121	5	Normal estrus behavior	No obvious stress pressure	2023/3/15	6.64	15.34
				2023/3/23	15.53	4.11
				2023/4/1	186.07	7.73
				2023/4/24	5.52	82.14
853	11	Normal estrus behavior	No obvious stress pressure	2023/3/14	3.66	11.02
				2023/3/26	10.25	8.16
				2023/4/5	165.65	12.36
				2023/4/26	2.47	55.41
870	10	Normal estrus behavior	No obvious stress pressure	2023/2/21	4.17	23.57
				2023/3/21	11.45	5.26
				2023/4/1	87.19	9.54
				2023/5/9	3.81	113.66
997	7	Normal estrus behavior	No obvious stress pressure	2023/1/16	2.62	22.29
				2023/2/11	13.56	1.26
				2023/2/22	200.09	8.04
				2023/3/2	5.80	44.49

561	20	Normal estrus behavior	No obvious stress pressure	2023/1/17	4.86	25.94
				2023/2/17	10.53	12.22
				2023/2/27	134.91	11.47
				2023/3/22	3.79	55.46
796	13	Normal estrus behavior	No obvious stress pressure	2023/1/25	4.08	15.21
				2023/2/24	20.04	7.86
				2023/3/8	136.00	7.91
				2023/3/22	4.04	49.02
598	18	Normal estrus behavior	No obvious stress pressure	2023/2/3	7.28	27.95
				2023/3/2	15.75	3.35
				2023/3/11	184.12	11.11
				2023/3/14	16.90	34.62
763	14	Normal estrus behavior	No obvious stress pressure	2023/2/24	5.46	18.43
				2023/3/24	14.33	14.89
				2023/4/2	133.67	9.20
				2023/4/24	5.03	107.95
801	13	Normal estrus behavior	No obvious stress pressure	2023/3/18	4.50	18.45
				2023/4/17	17.54	7.27
				2023/4/25	158.54	11.27
				2023/5/12	5.74	40.36
681	16	Normal estrus behavior	No obvious stress pressure	2023/3/18	5.30	30.61
				2023/4/17	13.09	9.32
				2023/4/25	129.42	9.83
				2023/5/24	2.04	62.72
