# Supplementary material for: Biochemical Characteristics of Urine Metabolomics in Female Giant Pandas at Different Estrous Stages
Source: Animals (Basel). 2024 Dec 3;14(23):3486. doi: 10.3390/ani14233486 (PMC11640436; doi:10.3390/ani14233486)
Supplement: Supplementary file 1 [file animals-14-03486-s001.zip › Table S2. The specific metabolites in Pos_cluster 2.pdf]

Table S2. The specific metabolites in Pos_cluster 2.

KEGG pathway	*P*-value	Metabolite
AMPK signaling pathway	0.018019788	Berberine / D-Fructose 1,6-bisphosphate
Regulation of actin cytoskeleton	0.032069971	Acetylcholine
Insulin secretion	0.032069971	Acetylcholine
Pancreatic secretion	0.032069971	Acetylcholine
Cholinergic synapse	0.063202223	Acetylcholine
Salivary secretion	0.063202223	Acetylcholine
Gastric acid secretion	0.063202223	Acetylcholine
Nicotine addiction	0.063202223	Acetylcholine
Carbon metabolism	0.06897058	D-Fructose 1,6-bisphosphate / Sedoheptulose 1,7-bisphosphate
Histidine metabolism	0.087784925	Carnosine / L-Histidinol
Longevity regulating pathway - worm	0.093421506	Nicotinamide
Glucagon signaling pathway	0.093421506	D-Fructose 1,6-bisphosphate
Neuroactive ligand-receptor interaction	0.108033214	Acetylcholine / Hydrocortisone
Synaptic vesicle cycle	0.122751987	Acetylcholine
Aldosterone-regulated sodium reabsorption	0.122751987	Hydrocortisone
Glycerophospholipid metabolism	0.178840369	Acetylcholine
Cortisol synthesis and secretion	0.178840369	Hydrocortisone
Cushing's syndrome	0.178840369	Hydrocortisone
Central carbon metabolism in cancer	0.178840369	D-Fructose 1,6-bisphosphate
Bile secretion	0.18719361	Acetylcholine / Hydrocortisone
