# Supplementary material for: Biochemical Characteristics of Urine Metabolomics in Female Giant Pandas at Different Estrous Stages
Source: Animals (Basel). 2024 Dec 3;14(23):3486. doi: 10.3390/ani14233486 (PMC11640436; doi:10.3390/ani14233486)
Supplement: Supplementary file 1 [file animals-14-03486-s001.zip › Table S3. The specific metabolites in Pos_cluster 5.pdf]

Table S3. The specific metabolites in Pos_cluster 5.

KEGG pathway	*P*-value	Metabolite
Vitamin digestion and absorption	0.04426606	Thiamine / Vitamin A / Vitamin B12
Sphingolipid metabolism	0.04664723	Phosphoethanolamine
Retinol metabolism	0.04664723	Vitamin A
ABC transporters	0.058337098	L-Histidines / Thiamine / Vitamin B12
Endocrine resistance	0.091248529	Testosterone
Sphingolipid signaling pathway	0.091248529	Phosphoethanolamine
Sulfur relay system	0.091248529	Thiamine
alpha-Linolenic acid metabolism	0.174646111	Methyl jasmonate
Thiamine metabolism	0.174646111	Thiamine
Dopaminergic synapse	0.174646111	3-Methoxytyramine
African trypanosomiasis	0.174646111	L-Kynurenine
Galactose metabolism	0.250826862	D-Tagatose
Glycerophospholipid metabolism	0.250826862	Phosphoethanolamine
Central carbon metabolism in cancer	0.250826862	L-Histidines
Steroid hormone biosynthesis	0.273677539	Androsterone / Testosterone
Prostate cancer	0.286395913	Testosterone
Vitamin B6 metabolism	0.32037706	4-Pyridoxic acid
Pathways in cancer	0.32037706	Testosterone
Taste transduction	0.352836663	D-Serine
Ovarian steroidogenesis	0.352836663	Testosterone
