# Supplementary material for: Biochemical Characteristics of Urine Metabolomics in Female Giant Pandas at Different Estrous Stages
Source: Animals (Basel). 2024 Dec 3;14(23):3486. doi: 10.3390/ani14233486 (PMC11640436; doi:10.3390/ani14233486)
Supplement: Supplementary file 1 [file animals-14-03486-s001.zip › Table S4. The specific metabolites in Neg_cluster 2.pdf]

Table S4. The specific metabolites in Neg_cluster 2.

KEGG pathway	*P*-value	Metabolite
Galactose metabolism	0.002212868	Dulcitol / Inositol / Sucrose
Cysteine and methionine metabolism	0.025005044	L-Homocystine / S-Adenosylhomocysteine / S-Sulfo-L-cysteine
Synthesis and degradation of ketone bodies	0.052478134	Acetoacetate
Phosphatidylinositol signaling system	0.052478134	Inositol
Carbohydrate digestion and absorption	0.052478134	Sucrose
Butanoate metabolism	0.075278008	Acetoacetate / Succinic semialdehyde
Steroid hormone biosynthesis	0.099544336	16-Glucuronide-estriol / Aldosterone / Androsterone glucuronide
Inositol phosphate metabolism	0.102347706	Inositol
Sulfur metabolism	0.149731112	Adenosine 5'-phosphosulfate
Cholesterol metabolism	0.149731112	Taurochenodeoxycholic acid
Nicotinate and nicotinamide metabolism	0.182155631	Quinolinic acid / Succinic semialdehyde
Pentose and glucuronate interconversions	0.194745347	L-Arabinitol
Valine, leucine and isoleucine degradation	0.194745347	Acetoacetate
Starch and sucrose metabolism	0.194745347	Sucrose
Aldosterone-regulated sodium reabsorption	0.194745347	Aldosterone
Caffeine metabolism	0.237502231	1-Methyluric acid
Propanoate metabolism	0.237502231	Acetoacetate
Fatty acid biosynthesis	0.278108621	Lauric acid
Primary bile acid biosynthesis	0.278108621	Taurochenodeoxycholic acid
Monobactam biosynthesis	0.278108621	Adenosine 5'-phosphosulfate
