# Supplementary material for: Biochemical Characteristics of Urine Metabolomics in Female Giant Pandas at Different Estrous Stages
Source: Animals (Basel). 2024 Dec 3;14(23):3486. doi: 10.3390/ani14233486 (PMC11640436; doi:10.3390/ani14233486)
Supplement: Supplementary file 1 [file animals-14-03486-s001.zip › Table S5. The specific metabolites in Neg_cluster 5.pdf]

Table S5. The specific metabolites in Neg_cluster 5.

KEGG pathway	*P*-value	Metabolite
C5-Branched dibasic acid metabolism	0.013101727	alpha-Ketoglutaric acid / Mesaconic acid
Taurine and hypotaurine metabolism	0.029814155	alpha-Ketoglutaric acid / L-Cysteinesulfinic acid
Glyoxylate and dicarboxylate metabolism	0.029814155	alpha-Ketoglutaric acid / Mesaconic acid
Butanoate metabolism	0.029814155	alpha-Ketoglutaric acid / Maleic acid
mTOR signaling pathway	0.063202223	5'-Adenylic acid
PI3K-Akt signaling pathway	0.063202223	5'-Adenylic acid
Lysine biosynthesis	0.093421506	alpha-Ketoglutaric acid
D-Glutamine and D-glutamate metabolism	0.093421506	alpha-Ketoglutaric acid
FoxO signaling pathway	0.093421506	5'-Adenylic acid
Olfactory transduction	0.093421506	5'-Adenylic acid
Pentose and glucuronate interconversions	0.122751987	alpha-Ketoglutaric acid
Riboflavin metabolism	0.122751987	Vitamin B2
cGMP-PKG signaling pathway	0.122751987	5'-Adenylic acid
Longevity regulating pathway	0.122751987	5'-Adenylic acid
Propanoate metabolism	0.151217262	2-Hydroxybutyric acid
Antifolate resistance	0.178840369	5'-Adenylic acid
Arginine biosynthesis	0.205643799	alpha-Ketoglutaric acid
Porphyrin and chlorophyll metabolism	0.205643799	Bilirubin
AMPK signaling pathway	0.205643799	5'-Adenylic acid
Renin secretion	0.205643799	5'-Adenylic acid
