# Supplementary figures and images for: Biochemical Characteristics of Urine Metabolomics in Female Giant Pandas at Different Estrous Stages
Source: Animals (Basel). 2024 Dec 3;14(23):3486. doi: 10.3390/ani14233486 (PMC11640436; doi:10.3390/ani14233486)

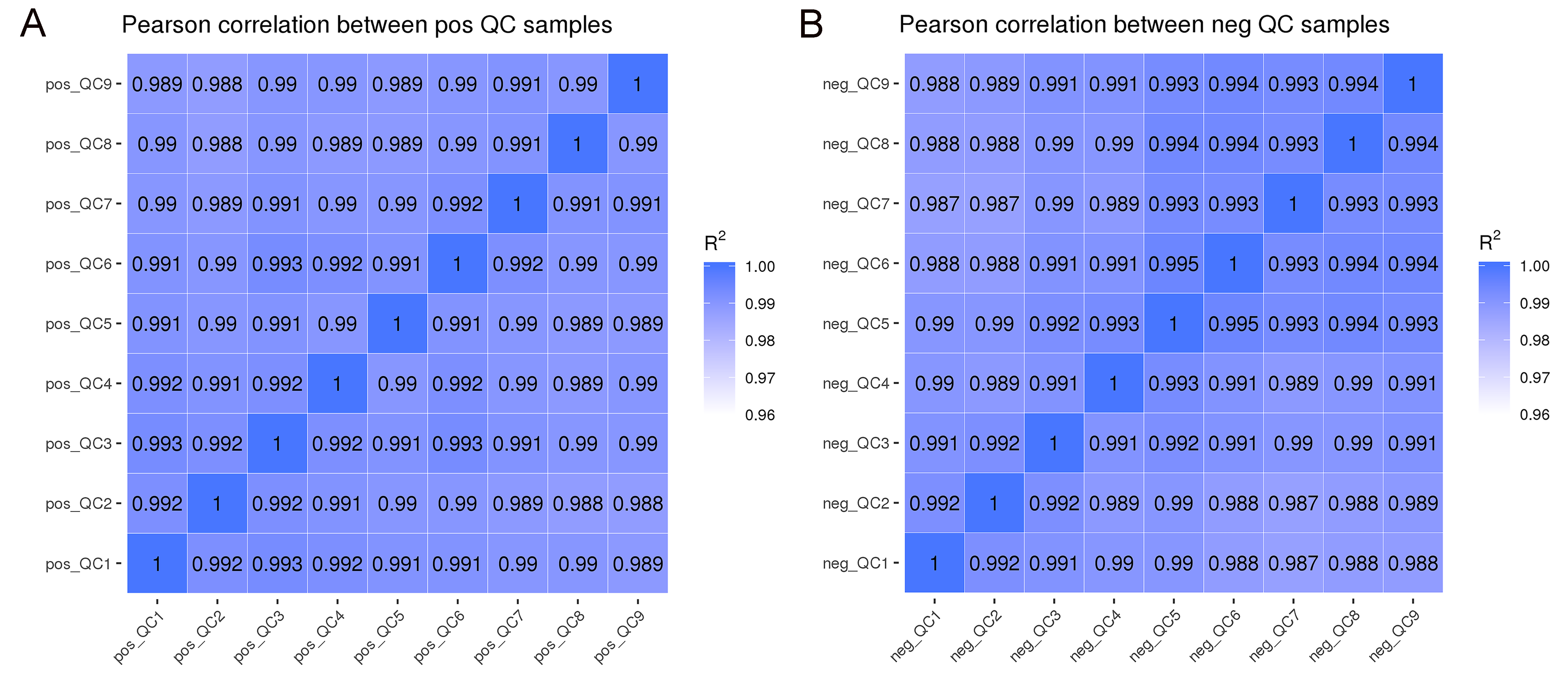

Supplement: Supplementary file 1 [file animals-14-03486-s001.zip › Figure S1.tif]
